# Supplementary material for: High Expression of SOD2 Protein Is a Strong Prognostic Factor for Stage IIIB Squamous Cell Cervical Carcinoma
Source: Antioxidants (Basel). 2021 May 5;10(5):724. doi: 10.3390/antiox10050724 (PMC8147985; doi:10.3390/antiox10050724)
Supplement: Supplementary file 1 [file antioxidants-10-00724-s001.zip › antioxidants-1179781-supplementary.pdf]

High Expression of SOD2 Protein Is a Strong Prognostic Factor for Stage IIIB Squamous Cell Cervical Carcinoma

Maria Cecília Ramiro Talarico [1], Rafaella Almeida Lima Nunes [2], Gabriela Ávila Fernandes Silva [2], Larissa Bastos Eloy Da Costa [3], Marcella Regina Cardoso [1], Sérgio Carlos Barros Esteves [4], Luis Otávio Zanatta Sarian [1], Luiz Carlos Zeferino [1†*], Lara Termini [2†*]

[1] Department of Obstetrics and Gynecology, Division of Gynecologic and Breast Oncology, School of Medical Sciences, State University of Campinas (UNICAMP – Universidade Estadual de Campinas), Campinas, São Paulo, Brazil; mcecilia_r@hotmail.com (M.C.R.T.); mrcardoso@mgh.harvard.edu (M.R.C.); sarian@unicamp.br (L.O.Z.S.); zeferino@unicamp.br (L.C.Z.)

[2] Centro de Investigação Translacional em Oncologia, Instituto do Câncer do Estado de São Paulo, Hospital das Clínicas da Faculdade de Medicina da Universidade de São Paulo, São Paulo, São Paulo, Brazil; rafaellaln30@gmail.com (R.A.L.N.); gabriela.avilafs@gmail.com (G.Á.F.S.); lara.termini@hc.fm.usp.br (L.T.)

[3] Department of Pathology, State University of Campinas (UNICAMP – Universidade Estadual de Campinas), Campinas, São Paulo, Brazil; larissa_eloy@yahoo.com.br (L.B.E.C.)

[4] Department of Radiotherapy, Division of Gynecologic and Breast Oncology, Women's Hospital Professor Doutor José Aristodemo Pinotti – Centro de Atenção Integral à Saúde da Mulher (CAISM), State University of Campinas (UNICAMP – Universidade Estadual de Campinas), Campinas, São Paulo, Brazil; sergiocb@unicamp.br (S.C.B.E.)

† These authors contributed equally to this work.

* Correspondence: zeferino@unicamp.br (L.C.Z.) - Phone: +55-19-3521-9516; lara.termini@hc.fm.usp.br (L.T.)

Supplementary Materials

Contents:

Table S1. The immunohistochemical positivity reaction performed using Image J®.

Table S2. Univariate and multivariate Cox regression analysis for disease-free survival and overall survival according to patient's age, tumor grade, treatment, and SOD2 expression as continuous variable (n= 55).

Figure S1: ROC curve discriminates the scores for SOD2 expression as predictor of recurrence.

Table S3: Sensitivity, specificity, positive predictive value, negative predictive value, and accuracy for cut-off score average ≥ 1.90 as a predictor of recurrence.

Figure S2: ROC curve discriminates the scores for SOD2 expression as predictor of death

Table S4: Sensitivity, specificity, positive predictive value, negative predictive value, and accuracy for cut-off score average ≥ 1.90 as a predictor of death.

Table S1. The immunohistochemical positivity reaction performed using Image J®

Patient Number	Field I			Field II			Field III			Score Average
	Ratio	Intensity	Score	Ratio	Intensity	Score	Ratio	Intensity	Score	
1	0.87	1	0.87	1	1	1	0.96	1	0.96	0.94
2	0	0	0	0	0	0	0	0	0	0.00
3	0.7	1	0.7	0.8	1	0.8	1	1	1	0.83
4	1	3	3	1	1	1	1	1	1	1.67
5	0.85	1	0.85	0.9	1	0.9	1	1	1	0.92
6	1	1	1	1	2	2	1	2	2	1.67
7	0	0	0	0	0	0	0	0	0	0.00
8	0	0	0	0	0	0	0	0	0	0.00
9	1	1	1	0.67	1	0.67	0.55	1	0.55	0.74
10	0	0	0	0	0	0	0	0	0	0.00
11	1	2	2	1	1	1	1	1	1	1.33
12	1	2	2	1	2	2	1	2	2	2.00
13	0	0	0	0	0	0	0	0	0	0.00
14	0.25	1	0.25	0.18	1	0.18	0.38	1	0.38	0.27
15	0.92	2	1.84	0.86	2	1.72	0.91	2	1.82	1.79
16	1	3	3	1	3	3	0.93	2	1.86	2.62
17	1	1	1	1	2	2	1	1	1	1.33
18	0	0	0	0	0	0	0	0	0	0.00
19	0.23	1	0.23	0.27	1	0.27	0.18	1	0.18	0.23
20	1	3	3	1	3	3	1	2	2	2.67
21	1	1	1	1	2	2	1	2	2	1.67
22	1	1	1	1	1	1	1	2	2	1.33
23	1	2	2	1	3	3	1	3	3	2.67
24	1	2	2	1	2	2	1	1	1	1.67
25	0.62	1	0.62	0.84	1	0.84	0.9	1	0.9	0.79
26	0.6	2	1.2	0.6	2	1.2	0.8	2	1.6	1.33
27	0.34	1	0.34	0.46	1	0.46	0.66	1	0.66	0.49
28	0.76	2	1.52	0.88	2	1.76	0.89	3	2.67	1.98

29	0.36	1	0.36	0.67	1	0.67	0.62	1	0.62	0.55
30	0.8	2	1.6	0.6	2	1.2	1	2	2	1.60
31	1	1	1	1	1	1	0.86	1	0.86	0.95
32	1	3	3	1	3	3	1	3	3	3.00
33	0.94	1	0.94	0.97	1	0.97	1	1	1	0.97
34	1	1	1	1	1	1	0	0	0	0.67
35	0.91	1	0.91	0.83	1	0.83	1	1	1	0.91
36	0.86	1	0.86	0.63	1	0.63	0.69	1	0.69	0.73
37	1	2	2	0.94	1	0.94	1	3	3	1.98
38	0.18	1	0.18	0.16	1	0.16	0.22	1	0.22	0.19
39	0.97	1	0.97	1	1	1	1	1	1	0.99
40	0	0	0	0	0	0	0	0	0	0.00
41	1	3	3	1	2	2	1	2	2	2.33
42	0.76	1	0.76	1	1	1	1	1	1	0.92
43	0.32	1	0.32	0.37	1	0.37	0.42	1	0.42	0.37
44	1	1	1	1	1	1	1	1	1	1.00
45	1	3	3	1	3	3	0.89	3	2.67	2.89
46	1	3	3	1	2	2	1	2	2	2.33
47	0.57	1	0.57	0.76	1	0.76	0.83	1	0.83	0.72
48	0.64	1	0.64	0.47	1	0.47	0.54	1	0.54	0.55
49	1	3	3	0.86	2	1.72	0.82	2	1.64	2.12
50	1	3	3	1	3	3	1	3	3	3.00
51	1	1	1	0.93	1	0.93	1	1	1	0.98
52	1	3	3	1	2	2	1	3	3	2.67
53	1	2	2	1	2	2	1	2	2	2.00
54	1	3	3	0.92	1	0.92	0.47	2	0.94	1.62
55	1	1	1	1	1	1	1	1	1	1.00

The count of the SOD2 positive cells and of the total number of cells per field were carried out in three fields to obtain the positive cell ratio in each case. Staining intensity was classified as 0 (no staining), 1 (weak), 2 (moderate), or 3 (strong). The stained cell ratio score and the staining intensity score were multiplied by each other to obtain a final score of each of the fields that were analyzed. A score average ranging between 0 and 3 was calculated.

Table S2. Univariate and multivariate Cox regression analysis for disease-free survival and overall survival according to patient's age, tumor grade, treatment, and SOD2 expression as continuous variable (n= 55).

**Univariate Analysis**	**Categories**	**DFS**		**OS**	
		**HR[#]**	**95% CI[#]**	**HR[##]**	**95% CI[##]**
SOD2 expression [α]	Cont.[¥]	1.60	1.00 – 2.55	1.43	0.89 – 2.29
**Multivariate Analysis**					
	<50	2.70	0.86 – 8.54	5.11	1.35 – 19.31
Age	50-59	1.00	-	1.00	-
	≥60	1.49	0.53 – 4.21	2.53	0.79 – 8.14
Anatomopathological	1 and 2	1.00	-	1.00	-
tumor grade [*]	3	1.34	0.42 – 4.29	1.63	0.50 – 5.35
Treatment	CRT	1.00	-	1.00	-
	RT	1.77	0.73 – 4.28	1.76	0.70 – 4.41
SOD2 expression [α]	Cont. [¥]	1.46	0.90 – 2.39	1.34	0.82 – 2.18

DFS = Disease-free survival. OS= Overall survival. [#] HR (hazard ratio) = Hazard ratio to relapse; censored = 32 patients; relapse= 23 patients. [##] HR (hazard ratio) = Hazard ratio to death; censored = 33 patients; death = 22 patients. CI= Confidence Interval. * Some cases lacked information on tumor grade, acute toxicity, and late toxicity; therefore, only cases with such information were presented. [α] SOD2 expression was translated into a score based on the ratio of stained cells and on staining intensity, then classified as low (<1.9) or high expression (≥1.9). [¥] Cont. = continuous variable.

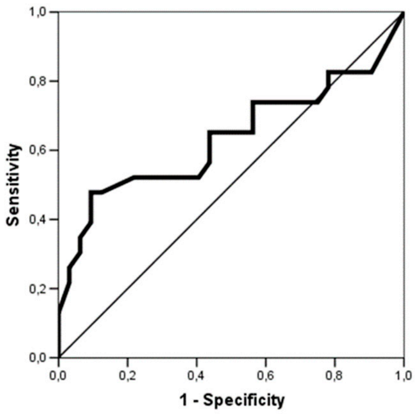

Figure S1: ROC curve discriminates the scores for SOD2 expression as predictor of recurrence. (AUC: 0.635; $p = 0.090$; 95% CI: (0.473–0.797); Cut-off: Score Average ≥ 1.90)

Table S3: Sensitivity, specificity, positive predictive value, negative predictive value, and for cut-off score average ≥ 1.90 as predictor of recurrence.

**Cut-off Score Average ≥ 1.90**	**%**	**95% CI**	
Sensitivity	47.83	(27.42–68.92)	Chi-square test: $\chi^2 = 10.43$; DF = 1; $\boldsymbol{p} = \mathbf{0.001}$
Specificity	90.63	(73.83–97.55)	
Positive predictive value	78.57	(48.82–94.29)	
Negative predictive value	70.73	(54.26–83.35)	
Accuracy	72.73	(58.81–83.46)	

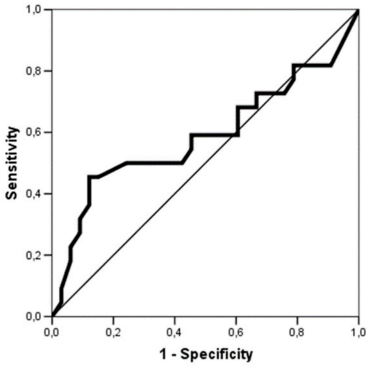

Figure S2: ROC curve discriminates the scores for SOD2 expression as predictor of death (AUC:0.588; $p = 0.272$; 95% CI: (0.421–0.755); Cut-off: Score Average ≥ 1.90)

Table S4: Sensitivity, specificity, positive predictive value, negative predictive value, and accuracy for cut-off score average ≥ 1.90 as predictor of death.

**Cut-off Score Average ≥ 1.90**	**%**	**95% CI**	
Sensitivity	45.45%	(25.07–67.33)	Chi-square test: $\chi^2 = 7.73$; DF = 1; $\boldsymbol{p} = \mathbf{0.005}$
Specificity	87.88%	(70.86–96.04)	
Positive predictive value	71.43%	(42.00–90.42)	
Negative predictive value	70.73%	(54.26–83.35)	
Accuracy	70.91%	(56.91–81.98)	
